# Liquid biopsy biomarkers to guide immunotherapy in breast cancer

**DOI:** 10.3389/fimmu.2023.1303491

**Published:** 2023-11-23

**Authors:** Jinghan Yang, Liang Qiu, Xi Wang, Xi Chen, Pingdong Cao, Zhe Yang, Qiang Wen

**Affiliations:** ^1^ Department of Biological Science, Vanderbilt University, Nashville, TN, United States; ^2^ Department of Radiation Oncology, Stanford University, Palo Alto, CA, United States; ^3^ Department of Computer Science and Engineering, The Chinese University of Hong Kong, Hong Kong, Hong Kong SAR, China; ^4^ Department of Human Resource, Shandong Provincial Hospital Affiliated to Shandong First Medical University, Jinan, China; ^5^ Department of Radiation Oncology, Shandong Provincial Hospital Affiliated to Shandong First Medical University, Jinan, China

**Keywords:** liquid biopsy, breast cancer, immunotherapy, immune checkpoint inhibitors, biomarkers

## Abstract

Immune checkpoint inhibitors (ICIs) therapy has emerged as a promising treatment strategy for breast cancer (BC). However, current reliance on immunohistochemical (IHC) detection of PD-L1 expression alone has limited predictive capability, resulting in suboptimal efficacy of ICIs for some BC patients. Hence, developing novel predictive biomarkers is indispensable to enhance patient selection for immunotherapy. In this context, utilizing liquid biopsy (LB) can provide supplementary or alternative value to PD-L1 IHC testing for identifying patients most likely to benefit from immunotherapy and exhibit favorable responses. This review discusses the predictive and prognostic value of LB in breast cancer immunotherapy, as well as its limitations and future directions. We aim to promote the individualization and precision of immunotherapy in BC by elucidating the role of LB in clinical practice.

## 1 Introduction

Over the past decade, cancer immunotherapy has emerged as an effective anti-tumor therapeutic approach on par with traditional modalities like chemotherapy, radiotherapy, and surgery. In particular, immune checkpoint inhibitors (ICIs) that target the programmed death-ligand 1 (PD-L1), programmed death 1 (PD-1), and cytotoxic T-lymphocyte-associated protein 4 (CTLA-4) pathways can restore T-cell functionality and promote anti-tumor immunity (1). As a result, ICIs including the anti-PD-L1 antibody atezolizumab (2), the anti-PD-1 antibody pembrolizumab (3), and the anti-CTLA-4 antibody ipilimumab (4), have been approved by the Food and Drug Administration (FDA) and European Medicines Agency (EMA) for various cancer types (5).

Breast cancer (BC) represents the leading cause of cancer-related mortality and most frequently diagnosed malignancy among women worldwide (6). Historically characterized as a ‘cold’ tumor type, BC exhibits a less inflammatory tumor microenvironment compared to ‘hot’ tumors with heightened immunogenicity and abundant tumor-infiltrating lymphocytes (TILs) (7). However, remarkable progress has been made with PD-1/PD-L1 agents in triple-negative breast cancer (TNBC), resulting in promising outcomes in both early (8, 9) and metastatic cases (10, 11). Moreover, ongoing research is actively investigating their potential in human epidermal growth factor receptor 2 (HER-2) positive and high-risk hormone receptor (HR)+ BC (12). Notable clinical trials, including Impassion 130 and Keynote 355, have demonstrated substantial benefits of ICIs for BC treatment (11, 13). A comprehensive overview of key studies evaluating ICIs efficacy in BC is summarized in **Table 1** (14–25).

**Table 1:** Summary of immunotherapy trails in breast cancer.

Trail	Subtype	Experimental vs. Control	Antibody/Cut-off	ORR	PFS	OS
Metastasis BC with ICI + Chemo						
IMpassion-130 (11)	TNBC	Atezolizumab + Nab-pac vs. PBO+ Nab-pac	VENTANA PD-L1 IHC SP142 IC:1%	58.9 vs. 42.6	7.5 vs 5.0	25.4 vs. 17.9
KEYNOTE-355 (13)	TNBC	Pembrolizumab + Nab-pac (Pac or Gem-Carbo) vs. PBO + Nab-pac (Pac or Gem-Carbo)	Agilent PD-L1 IHC 22C3 CPS:1	53.2 vs. 39.8	9.7 vs. 5.6	23.0 vs. 16.1
IMpassion-131 (14)	TNBC	Atezolizumab + Pac vs. PBO + Pac	VENTANA PD-L1 IHC SP142 IC:1%	63.4 vs. 55.4	6.0 vs. 5.7	22.1 vs. 28.3
ENHANCE 1 (15)	HR+HER2+	Pembrolizumab + Eribulin vs. Eribulin	Agilent PD-L1 IHC 22C3 CPS:1	27.0 vs. 34.0	4.1 vs. 4.2	13.4 vs. 12.5
KELLY (16)	HR+HER2+	Pembrolizumab + Eribulin	Agilent PD-L1 IHC 22C3 CPS:1	40.9	6.0	59.1% for 1-year OS
Metastasis BC with ICI						
KEYNOTE-086 Cohort A (17)	TNBC	Pembrolizumab	Agilent PD-L1 IHC 22C3 CPS:1	5.3	2	9
KEYNOTE-086 Cohort B (17)	TNBC	Pembrolizumab	Agilent PD-L1 IHC 22C3 CPS:1	21.4	2.1	18
KEYNOTE-119 (18)	TNBC	Pembrolizumab vs. TPC	Agilent PD-L1 IHC 22C3 CPS:1	9.6 vs. 10.6	2.1 vs. 3.3	9.9 vs. 10.8
KEYNOTE-028 (19)	HR+HER2+	Pembrolizumab	Agilent PD-L1 IHC 22C3 CPS:1	12	1.8	8.6
JAVELIN (20)	TNBC	Avelumab	Dako PD-L1 IHC 73-10 pharmDx; tumor cell: 1,5,25%; tumor associated cell:10%	5.2	1.5	9.2
JAVELIN (20)	HR+HER2+	Avelumab	Dako PD-L1 IHC 73-10 pharmDx; tumor cell: 1,5,25%; tumor associated cell:10%	2.8	NA	NA
NCT01375842 (21)	TNBC	Atezolizumab	VENTANA PD-L1 IHC SP142 IC:1%	10	1.4	8.9
Early Stage BC						
KEYNOTE-522 (22)	TNBC	Pembrolizumab + Cab + Pac vs. PBO+ Cab + Pac	Agilent PD-L1 IHC 22C3 CPS:1	64.8 VS.51.2	NA	NA
I-SPY 2 (23)	TNBC	Pembrolizumab + Cab vs. Pac	NA	60 vs 20	NA	NA
I-SPY 2 (23)	HR+ HER2+	Pembrolizumab + Cab vs. Pac	NA	30 vs. 13	NA	NA
IMpassion-031 (24)	TNBC	Atezolizumab + Nab-pac (AC) vs. PBO + Nab-pac (AC)	VENTANA PD-L1 IHC SP142 IC:1%	57.6 vs 41.1	NA	NA
GeparNuevo (9)	TNBC	Durvalumab+ Nab-pac vs. PBO+ Nab-pac	VENTANA PD-L1 IHC SP263 IC:1%	53.4 vs. 44.2	NA	NA
NeoTRIP (25)	TNBC	Atezolizumab + Carbo + Nab-pac vs. Carbo + Nab-pac	VENTANA PD-L1 IHC SP142 IC:1%	43.5 vs. 40.8	NA	NA

Currently, identifying appropriate first-line immunotherapy candidates within BC and predicting individual patient treatment responses primarily relies on immunohistochemistry (IHC) testing to evaluate PD-L1 expression levels. However, the utilization of PD-L1 as a sole biomarker and predictor encounters certain limitations and challenges. First, numerous different PD-L1 antibodies are currently employed for IHC-based tumor PD-L1 expression assessment, including Dako 28-8 rabbit monoclonal, Dako 22C3 mouse monoclonal, Roche Ventana SP142 rabbit monoclonal, and Roche Ventana SP263 rabbit monoclonal antibodies, introducing inherent variability into the PD-L1 results obtained from different studies and clinical settings (26). Second, the heterogeneity in IHC cutoff values for defining PD-L1 positivity across clinical trials utilizing different assay platforms leads to discrepancies in PD-L1 designation. Moreover, intratumoral heterogeneity of PD-L1 expression potentially underestimates overall PD-L1 status in the context of small tumor biopsy samples, which may not fully and accurately represent the entire heterogeneous PD-L1 expression profile within the tumor as a whole (27). Lastly, the predictive value of IHC-based PD-L1 expression for immunotherapy response is not definitive, due in part to practical challenges obtaining adequately sized and preserved tumor tissue samples and isolating sufficient quantities of viable tumor cells from limited biopsy specimens (28). Consequently, a subset of patients with PD-L1 positive tumors still lack significantly favorable clinical immunotherapy responses, necessitating the development and validation of additional robust predictive biomarkers to more precisely select candidates likely to derive maximal therapeutic benefit from ICIs.

**Figure 1:**
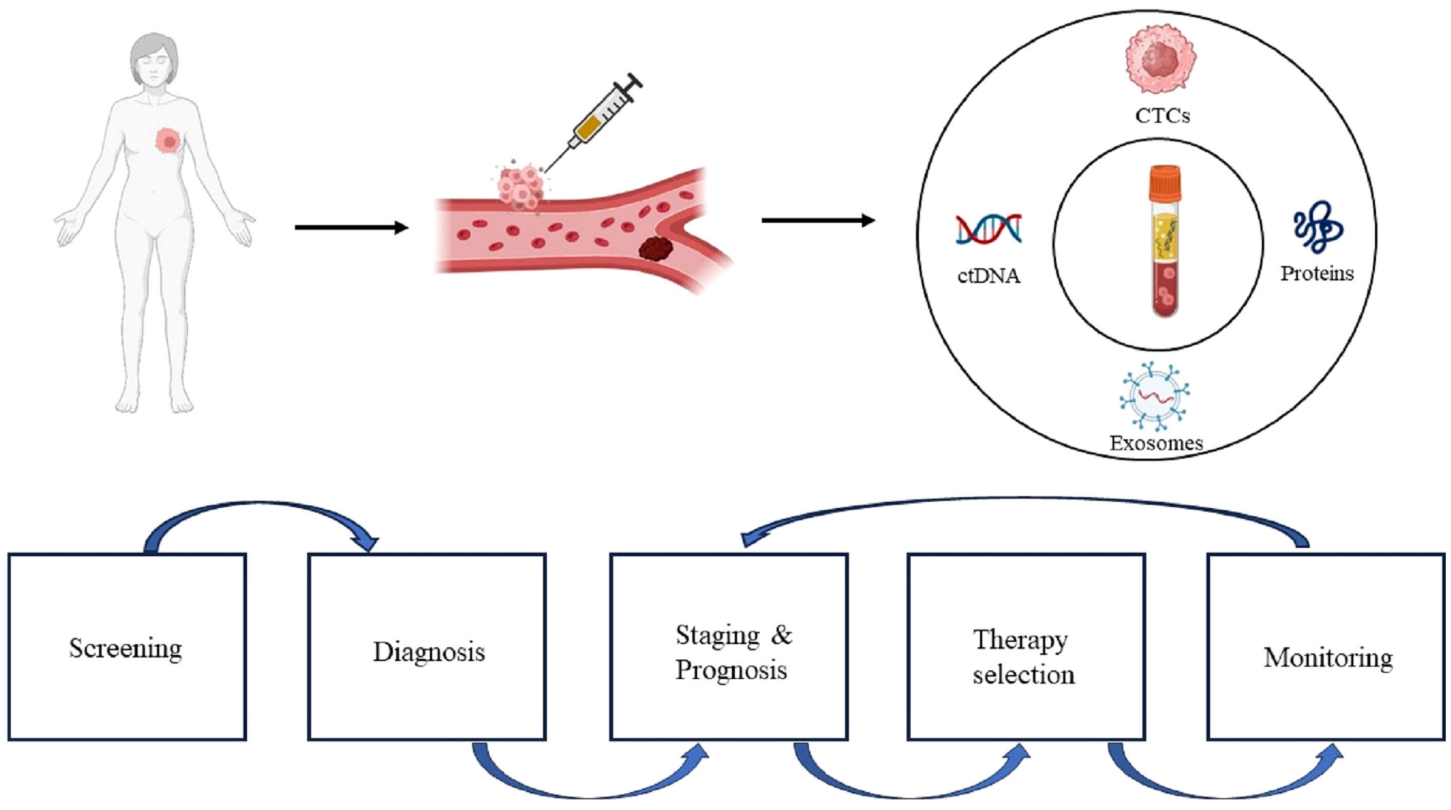
Clinical applications of liquid biopsy. The immunology information extracted from liquid biopsy can be used for continuous monitoring, from early stage disease screening, assistance diagnosis, personalized therapy selection, to recurrence monitoring. CTCs, circulating tumor cells; ctDNA, circulating tumor DNA.

**Figure 2:**
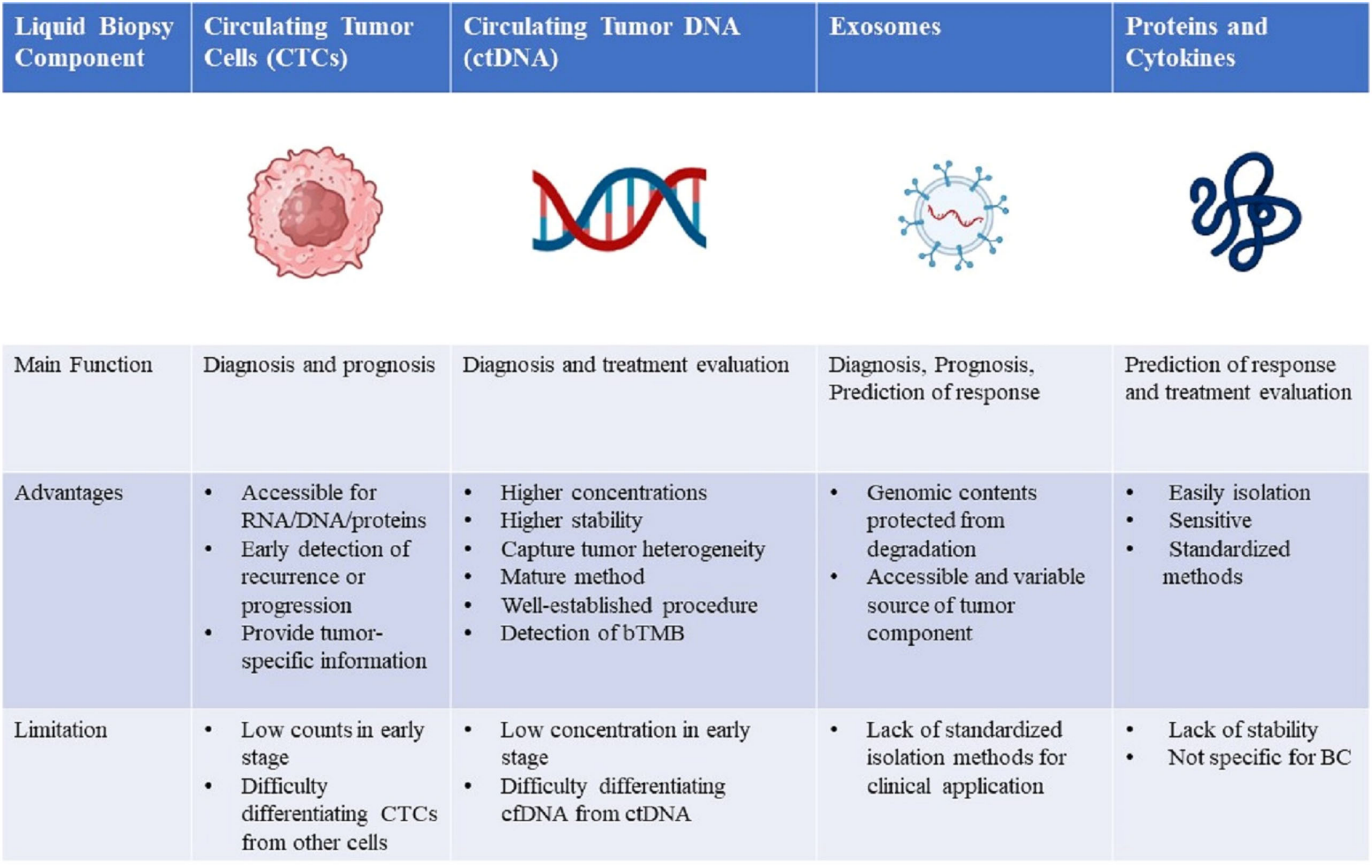
Comparison of four liquid biopsy components and the main advantages, disadvantages, and future directions of their clinical application in breast cancer management. RNA, ribonucleic acid; DNA, deoxyribonucleic acid; CTCs, circulating tumor cells; ctDNA, circulating tumor DNA; bTMB, blood-based tumor mutational burden; cfDNA, cell-free DNA; BC, breast cancer.

Liquid biopsy (LB) has recently emerged as a promising minimally invasive surrogate biomarker to guide immunotherapy decisions in BC. LB allows assessment of various tumor components in the peripheral blood, including circulating tumor cells (CTCs), circulating tumor DNA (ctDNA), exosomes, and proteins (**Figure 1**). Compared with traditional tissue biopsy, LB offers advantages such as minimal invasiveness, reproducibility, and rapid turnaround (**Figure 2**) (29). This review provides an updated overview of LB applications for ICIs therapy in BC, highlighting current research and future directions. We discuss the strengths and limitations of LB as a biomarker for BC immunotherapy, including its potential to identify responders, detect resistance mechanisms, and predict clinical outcomes. Ongoing studies will help validate the clinical utility of LB-based biomarkers to optimize patient selection and management for ICIs treatment in BC.

## 2 CTCs

CTCs present in the peripheral blood can be identified through analytical methods based on biological (e.g. epithelial markers and absent hematopoietic markers) and physical (e.g. size, density, invasiveness) characteristics (30–33). The CellSearch*™* system remains the only FDA-approved platform for CTCs detection in metastatic breast cancer to date (34). This system isolates EpCAM+ CTCs using antibody-coated magnetic beads, followed by immunofluorescent staining for cytokeratins and cluster of differentiation 45 (CD45) to distinguish CTCs from leukocytes (34).

Multiple studies have explored associations between CTCs and the tumor immune microenvironment (TIM) in breast cancer. For instance, patients with detectable CTCs were found to exhibit increased regulatory T cell infiltration in tumor tissues compared to those without CTCs, indicating an immunosuppressive phenotype (35). Mego et al. revealed an inverse correlation between CD8+ cytotoxic T cell levels and CTC counts in breast cancer tissues through immunohistochemical analysis. They also observed reduced dendritic cell infiltration into bone marrow metastatic niches accompanied by high CTC numbers in inflammatory breast cancer patients (36). Most recently, the same group demonstrated a positive correlation between mesenchymal-like CTCs undergoing epithelial-to-mesenchymal transition (EMT) and PD-L1 positive stromal cells in the tumor microenvironment (37). Together, these findings position CTCs as promising indicators of anti-tumor immune activity and immunosuppression within tumor tissues.

Recent technological advances have enabled comprehensive functional profiling of CTCs, providing powerful tools to identify predictive biomarkers for ICIs therapy in breast cancer. Specifically, single-cell proteomic, transcriptomic and metabolomic analyses of CTCs can elucidate multidimensional molecular characteristics associated with therapeutic response. For instance, proteomic profiling may reveal specific CTC subpopulations correlated with immunotherapeutic sensitivity or resistance. Transcriptomic sequencing could uncover distinct CTC gene signatures related to immune evasion mechanisms. Metabolomic analyses of CTCs may also provide insights into immunometabolic phenotypes influencing immunotherapy efficacy. Furthermore, the C-X-C chemokine receptor type 4 (CXCR-4) was found to be upregulated on breast cancer CTCs, suggesting a potential role in regulating immune cell recruitment and function in the tumor microenvironment during treatment (38). Assessing dynamic changes in CXCR4 expression on CTCs by single-cell assays may thus help monitor immune modulation effects. In summary, technological progress has enabled in-depth interrogation of CTCs as a valuable biomarker source to predict and monitor immunotherapy outcomes in breast cancer patients.

## 3 ctDNA

ctDNA is released into the bloodstream by tumor cells, distinguishing it from cell-free DNA (cfDNA) derived from normal apoptotic or necrotic cells. Compared to CTCs, ctDNA is present at higher concentrations in plasma, making it an attractive noninvasive liquid biopsy target (39). Somatic genomic alterations specific to cancer cells enable the differentiation of tumor-derived ctDNA from normal cfDNA in blood (40). ctDNA holds promise as a biomarker for early detection of metastasis and disease recurrence post-treatment (41, 42). However, clinical data on the utility of ctDNA to predict immunotherapy outcomes remains limited thus far.

A study by Magbanua et al. analyzed 511 plasma samples from 138 high-risk HR+/HER2- breast cancer patients who received pembrolizumab with neoadjuvant chemotherapy in the I-SPY2 trial (43). ctDNA levels declined over time in both the pembrolizumab and control arms. All patients achieving pathological complete response (pCR) cleared ctDNA prior to surgery. Among non-pCR patients, those ctDNA-negative after neoadjuvant treatment exhibited markedly higher distant recurrence-free survival compared to ctDNA-positive patients, with a hazard ratio (HR) of 0.13. This supports the potential of longitudinal ctDNA monitoring to guide clinical decision-making in breast cancer immunotherapy. Additionally, the INSPIRE trial by Bratman et al. prospectively assessed ctDNA dynamics as a biomarker of tumor burden in diverse cancer patients on pembrolizumab (44). Cohorts included head and neck squamous cell carcinoma (HNSCC), TNBC, ovarian cancer, melanoma, and mixed solid tumors (MST). Patients with decreased ctDNA after 3 treatment cycles had improved clinical benefit rate (CBR), overall survival (OS), and progression-free survival(PFS); whereas increased ctDNA indicated disease progression and poorer survival (median OS 13.7 months). Undetectable ctDNA levels strongly correlated with therapeutic response. Importantly, ctDNA changes provided complementary data to the Response Evaluation Criteria in Solid Tumors (RECIST) criteria for predicting immunotherapy survival benefit. Overall, detecting ctDNA dynamics noninvasively predicts immunotherapy outcomes and has implications for guiding breast cancer treatment.

In addition to tumor burden monitoring, ctDNA analysis can provide insight into immunotherapy response mechanisms. Somatic mutation profiling of ctDNA may reveal neoantigen loss associated with acquired resistance. Integrated genomic and transcriptomic ctDNA data may elucidate immune evasion pathways in breast cancer immunotherapy. Emerging techniques like low-pass whole genome sequencing help overcome technical hurdles in detecting scarce ctDNA.

## 4 Exosomes

Extracellular vesicles (EVs) are an integral intercellular communication strategy utilized by both pathogenic and non-pathogenic cells (45). Considerable evidence indicates EVs play a key role in the interaction between tumor cells and immune cells (46). Tumor-derived EVs predominantly demonstrate immunosuppressive capabilities, thereby promoting immune evasion of tumors (47). Such immunosuppression involves EVs downregulating major histocompatibility complex II (MHC II) expression in dendritic cells (48), activating cGAS-STING signaling in dendritic cells (49), inducing STAT3-mediated M2 polarization in monocytes (50), reducing interferon-gamma (IFN-γ) production in natural killer cells (51), and triggering apoptosis in T cells (52). Presentation of PD-L1 on EVs surfaces, as described above, induces T cell exhaustion and dampens anti-tumor immune responses.

Conversely, tumor-derived EVs have been shown to elicit immune activation by stimulating natural killer cells, macrophages, and B and T lymphocytes. Robust tumor clearance associated with EVs affecting the Hippo pathway has been linked to immune activation. EVs from antigen-presenting cells frequently exhibit immunostimulatory properties by carrying MHC complexes that activate T cells, although T cell stimulation by EVs is less potent (53).

Regarding the potential of EVs as biomarkers in immuno-oncology, increased vesicular PD-L1 in melanoma patients undergoing ICIs signifies adaptive immune responses and distinguishes clinical responders from non-responders (53). Elevated vesicular PD-L1 and CD28 correlate with improved PFS and OS in patients receiving anti-CTLA-4 therapy (54). Moreover, specific vesicular RNA profiles have been found to correlate with responses to anti-CTLA-4 treatment. In summary, EVs are integral immune modulators and profiling circulating EVs exhibits tremendous promise as a marker of immunogenicity. While profiling EVs in the blood of breast cancer patients is commonplace, major hurdles remain regarding validated, standardized isolation techniques. Resolving these issues is an imperative first step toward clinically implementing EV analysis.

## 5 Proteins

### 5.1 PD-L1

The immune checkpoint protein PD-L1, also termed CD247 or B7-H1, is expressed on antigen presenting and tumor cells. Ligation of PD-L1 with its cognate receptor PD-1 found on T lymphocytes leads to inhibition of T-cell activation, resulting in impaired anti-cancer immunity. Monoclonal antibodies blocking the PD-1/PD-L1 axis have exhibited clinical activity in patients with elevated PD-L1 levels quantified through IHC staining of tumor biopsies. However, some individuals with low tumoral PD-L1 expression have also shown benefits from immune checkpoint blockade (55). This discrepancy is attributed to the dynamic features of immune regulation that cannot be fully captured in static IHC-based assessments. Moreover, PD-L1 expression on both malignant cells and infiltrating leukocytes creates challenges in interpreting PD-L1 levels in tumor tissues. Variations in PD-L1 detection antibodies remain an unresolved issue precluding standardization of PD-L1 IHC methodology. Finally, heterogeneous PD-L1 expression among primary and metastatic lesions restricts the utility of tissue-based approaches. Blood-based profiling of PD-L1 status through analyses of circulating markers like CTCs, EVs, peripheral blood mononuclear cells (PBMCs) could help overcome certain limitations inherent to tissue biopsies (**Table 2**).

**Table 2:** Studies on PD-L1 in breast cancer patients receiving immunotherapy.

Research	Subtype	Location	Sample size	Results
Mazel et al. (56)	HR+,HER2- metastatic BC	CTC	16	A strong heterogeneity in PD-L1 CTC expression, ranging from 0.2 to 100
Schott et al. (57)	Metastatic and early stage BC	CTC	72	More PD-L1+CTC in metastatic patients than patients without metastatic (75% vs. 61.1%)
Jacot et al. (58)	Metastatic BC	CTC	72	PD-L1+ CTCs was associated with PDS while tissue PD-L1 was not
Agelaki et al. (59)	Metastatic BC	CTC	98	PD-L1+ CTC was associated with shorter PFS(5.8 vs. 13.3m)and reduced OS(23.8 vs 35.7m)
Yang et al. (60)	TNBC cell line	EV	NA	Exosomes enhance anti-tumor immunity in PD-L1 downregulated tumor microenvironment; exosomes transfer PD-L1 from the positive cells to negative cells.
Li et al. (61)	TNBC	Soluble	66	sPD-1 was elevated in TNBC; a decrease in sPD-L1 levels were detected in patients with CR and PR

### 5.2 PD-L1 on CTCs

Expression of PD-L1 on tumor cells can be readily influenced by inflammatory, microenvironmental, and treatment-associated factor (56). Since CTCs arise from multiple tumor sites, they may better capture the heterogeneity of PD-L1 expression compared to localized tissue samples. Initial studies have established CTCs analysis as a platform to evaluate PD-L1 status in cancer patients. Mazel et al. performed the first study enumerating PD-L1-positive CTCs in metastatic breast carcinoma, revealing substantial variability with positivity ranging from 0.2-100% among 11/16 PD-L1-positive cases (57). This seminal study provided the foundation for subsequent research on PD-L1 expression in CTCs. Additional investigations have confirmed detection of PD-L1-positive CTCs in breast cancer and elucidated clinical implications. Schott et al. examined 72 breast cancer patients, identifying PD-L1-positive CTCs in 94.5% (57). Metastatic patients exhibited significantly higher CTC counts versus non-metastatic cases. (75% vs. 61.1%; p<0.05). Moreover, declining PD-L1-positive CTCs associated with treatment responses, indicating CTCs may serve as pharmacodynamic markers of immunotherapy efficacy. Interestingly, PD-L1-positive CTCs increased even after discontinuing ICIs, implying the ability of these inhibitors to reduce the quantity of PD-L1-positive CTCs in BC patients. Hence, PD-L1-positive CTCs presence associated with poorer prognosis and could be utilized to monitor immunotherapy efficacy while also reflecting potential resistance mechanisms.

In a prospective study, Jacot et al. detected CTCs and PD-L1-positive CTCs and PD-L1-positive CTCs pre-treatment in 79.2% and 36.1% of metastatic BC patients, respectively (59). Compared to tissue PD-L1 expression, PD-L1-positive CTCs were associated with shorter progression-free survival, although this was not confirmed on multivariate analysis. Compared to tissue PD-L1 expression, PD-L1-positive CTCs were associated with shorter progression-free survival, although this was not confirmed on multivariate analysis. Moreover, Compared to tissue PD-L1 expression, PD-L1-positive CTCs were associated with shorter progression-free survival, although this was not confirmed on multivariate analysis (62). High PD-L1 expression occurred in approximately 11.6% of patients and was associated with poorer median survival (23.8 vs 35.7 months, p=0.043). The study also demonstrated a significant correlation between PD-L1-positive CTCs and increased recurrence risk (HR = 4.8; p=0.011). These findings suggest that subgroups of BC patients with PD-L1-positive CTCs may derive greater benefit from anti-PD-L1 immunotherapy.

PD-L1-positive CTCs have also been confirmed in other malignancies, including non-small cell lung cancer (NSCLC) (63), head and neck cancer (64), colon cancer (65), prostate cancer (66), and pancreatic cancer (67). Recent comprehensive analysis of CTCs in breast cancer confirms similar patterns of PD-L1 and CD47 expression as seen in lung cancer. Papadaki et al. examined PBMCs from early stage and metastatic BC patients using triple immunofluorescence staining (68). PD-L1 enables immune evasion while CD47 signals “do not eat me” to macrophages. A lower concordance in PD-L1 and CD47 labeling between CTCs and tumor tissue as well as between PBMCs and TILs. Approximately 11-30% of CTCs were PD-L1/CD47 positive, increasing with disease progression. Critically, metastatic patients with high CD47/PD-L1 CTCs showed associations with poorer outcomes including shorter progression-free survival and greater risk of relapse and death. These data strengthen the biological rationale for dual PD-L1/CD47 inhibition in BC.

Liquid biopsy represents an advanced method for dynamically and continuously monitoring PD-L1 expression in breast cancer patients receiving immune checkpoint inhibitors. Further research is warranted on utilizing PD-L1-positive circulating tumor cells during immunotherapy and correlating their expression with tumor tissue. Before employing liquid biopsy for treatment decision-making, several issues must be addressed. These include the substantial evidence linking epithelial-mesenchymal transition and PD-L1 expression (69, 70), and the need to mitigate false-positive results since PD-L1 is also expressed on suppressor cells from the bone marrow (71, 72).

### 5.3 PD-L1 on exosomes

The clinical prognostic value of PD-L1 expression on exosomes has been validated in several solid tumor types (60, 73, 74) However, further studies are still needed to evaluate the clinical utility of exosomal PD-L1 specifically in breast cancer. Experimental findings have proposed that PD-L1 bound to the surface of exosomes can effectively interact with PD-1 receptors, resulting in inhibition of T cell activation, suppression of apoptosis in breast cancer cells, and facilitation of tumor immune evasion (61). Additionally, exosomes were able to transfer PD-L1 from PD-L1-positive cancer cells to PD-L1-negative cancer cells, elucidating the underlying mechanisms of immune evasion employed by breast cancer cells.

### 5.4 PD-L1 in plasma

Plasma represents another important specimen for liquid biopsy to detect PD-1, PD-L1, and PD-L2 (75). A notable study in 66 patients with TNBC revealed significantly higher plasma PD-L1 levels compared to healthy controls (76). Furthermore, serum PD-L1 levels correlated with tumor stage (p=0.030). Patients who achieved complete or partial response after neoadjuvant chemotherapy (NAC) exhibited decreased plasma PD-L1 levels, whereas patients with stable disease or disease progression displayed increased plasma PD-L1 levels. These findings demonstrate the potential clinical utility of measuring PD-L1 in plasma as a liquid biopsy approach for prognostication, predicting response to chemotherapy, and monitoring disease status in TNBC.

### 5.5 PD-L1 in PBMCs

Analysis of PBMCs from BC patients showed PD-L1 promoter hypomethylation may explain increased PD-L1 expression in PBMCs versus matched tumor tissue. Additionally, the PD-1 promoter was hypermethylated in PBMCs compared to tumor (77). Methylation profiling in cell-free DNA could thus serve as a molecular correlate for PD-L1 expression. Another study found significantly more PD-1 high CD8+ exhausted T cells in tumor versus matched blood of triple negative BC patients (78). These data demonstrate differential tumor immune interactions in circulation versus tissue.

## 6 Genomic biomarkers

### 6.1 Tumor mutational burden

Although the FDA and EMA no longer endorse tumor mutational burden (TMB) as a standard treatment selection biomarker, TMB remains a potential indicator of T-cell activation that may help predict response to ICIs therapy (79). While tumor tissue biopsies were previously the primary TMB sample source (80), alternative liquid biopsy samples like ctDNA and CTCs represent promising substitutes for TMB quantification in patients with limited tumor tissue.

Assessment of TMB from ctDNA represents a promising advancement that expands its application to patients with limited biopsy samples or difficulties obtaining high-quality tissue samples for TMB assays (81). Previous studies have demonstrated a correlation between blood-based TMB and tissue-based TMB (82, 83).

In one study of 30 patients, detectable mutations ranging from 1 to 53 were identified in ctDNA. Furthermore, decreased variant allele frequencies of ctDNA mutations were observed in 3 patients who had objective responses to treatment, suggesting ctDNA may enable early prediction of treatment efficacy. Gandara et al. analyzed two large retrospective randomized trials and showed a blood-based TMB threshold ≥16 was predictive of efficacy for ICIs therapy (84). These results indicated blood-based TMB (bTMB) could independently predict clinical benefit in terms of progression-free survival associated with atezolizumab. Use of plasma, rather than tissue, as a DNA source for assessing bTMB provides an attractive alternative for patients with metastatic non-small cell lung cancer who may not be suitable candidates for biopsy or lack sufficient tumor tissue.

In metastatic TNBC, the median value of biopsy-based TMB has been associated with breast tumor subtype and sample type. Higher TMB detected in tumor tissue was correlated with longer PFS, compared to bTMB (85, 86). Therefore, there are still certain challenges that need to be addressed and clarified regarding discordance between tissue and blood TMB. The establishment of standardized processes and meaningful thresholds would facilitate accurate assessment, taking into account the specific panel of genes that contribute significantly to the precise evaluation of bTMB (87). While a close correlation between tissue-based TMB and bTMB exists, bTMB is a relatively independent predictive factor (88–90). While a close correlation between tissue-based TMB and bTMB exists, bTMB is a relatively independent predictive factor.

### 6.2 dMMR/MSI

DNA mismatch repair (MMR) plays a vital role in maintaining DNA integrity by correcting errors during replication, recombination, and repair (91). MMR deficiencies can result in microsatellite instability (MSI), observed across cancer types. In colorectal cancer, increased mutational burden from deficient MMR (dMMR) and MSI associates with improved response to PD-1/PD-L1 blockade (92), leading to FDA approval of pembrolizumab for any dMMR/MSI tumor (93).

Despite the relatively low 1-2% incidence, current evidence remains insufficient regarding MSI/dMMR predictive value in breast cancer (94, 95). However, data indicate MSI presence across breast cancer subtypes, particularly in high grade, low progesterone receptor tumors (96). Cases showed metastatic breast cancer patients exhibited favorable immune checkpoint inhibitor responses, including nivolumab in dMMR/MSI triple negative breast cancer and pembrolizumab in dMMR/MSI luminal (97) or HER2+ disease with high tumor mutational burden and dMMR (98). Thus, utilizing dMMR as a predictive biomarker may improve outcomes and guide appropriate immune therapy selection.

Similarly, MSI evaluation can be performed via circulating tumor DNA analysis (99). Notably, MSI is effectively detected even at low coverage (100). Previous studies show high MSI levels in ctDNA correlate with improved immune checkpoint inhibitor responses (101). Detecting ctDNA somatic mutations may identify non-responders, since such mutations regulate tumor immunity. In anti-PD-1 treated pan-cancer cohorts, high pretreatment plasma MSI and tumor mutational burden strongly predicted progression-free survival (p=0.001 and 0.003, respectively) (102).

### 6.3 TCR repertoire

The clinical efficacy of ICIs relies on the recognition of neoantigens by T cells. These neoantigens are presented to T cells through interaction with MHC molecules (103). Appropriate T cell receptors (TCRs) recognize these neoantigens, triggering an immune response as they are perceived as foreign rather than self-antigens (104). Analysis of the TCR repertoire by sequencing the TCR CD3 region provides valuable insight, as the CD3 region is unique to each TCR, and its diversity can serve as a predictive biomarker for ICIs response (105).

One study reported the circulating CD8+ T cell TCR repertoire in the blood of breast cancer patients changed following chemotherapy (106). There was an association between increased TCR repertoire diversity and improved treatment outcomes. Gao et al. performed TCR sequencing on PBMCs from metastatic inflammatory and triple-negative breast cancer patients (107). Therefore, TCR sequencing from blood not only reflects the diversity of the TCR repertoire, but also serves as a surrogate indicator for evaluating the effectiveness of breast cancer immunotherapy.

## 7 Novel liquid biopsy approaches

CTCs and cfDNA in blood represent emerging liquid biomarkers with potential clinical utility for cancer management. In addition to detecting mutations in ctDNA, other novel cfDNA analysis approaches that go beyond mutation profiling are being developed and show promise.

One such approach is evaluation of genome-wide fragmentation patterns of cfDNA, termed “fragmentomics” (108). By combining fragmentation pattern analysis with mutation profiling, this approach can accurately discriminate between cancer patients and healthy individuals based on differences in cfDNA fragmentation profiles. Another emerging technique is methylation sequencing of cfDNA (109). For example, detailed evaluation of methylation patterns across more than 900 CpG sites in cfDNA has been shown to enable detection of cancer presence as well as identification of cancer type in patients with advanced cancers.

Chromatin state analysis and nucleosome footprinting of cfDNA are other approaches under development (110). Nucleosome positions on DNA determine chromatin structure, which in turn affects gene expression. These techniques involve generating genome-wide maps showing nucleosome occupancy and transcription factor binding patterns in cfDNA fragments. Analysis of such nucleosome footprints has revealed patient- and tumor-specific patterns that allow accurate prediction of cancer subtypes (111).

A key challenge is that tumor-derived DNA represents only a small fraction of total cfDNA. Tumors with low mutational burden like breast cancer are especially difficult to detect. However, these emerging cfDNA analysis platforms allow interrogation of significantly more genomic loci compared to targeted mutation panels. For example, low-coverage genome sequencing of cfDNA to measure copy number changes can monitor immunotherapy response (112). Such whole-genome analysis approaches complement mutation profiling and may provide clinically actionable information beyond what can be achieved with ctDNA analysis alone. Further validation of the ability of these novel platforms to guide immunotherapy decisions in cancers including breast cancer is warranted.

## 8 Challenge and future

**Figure 3:**
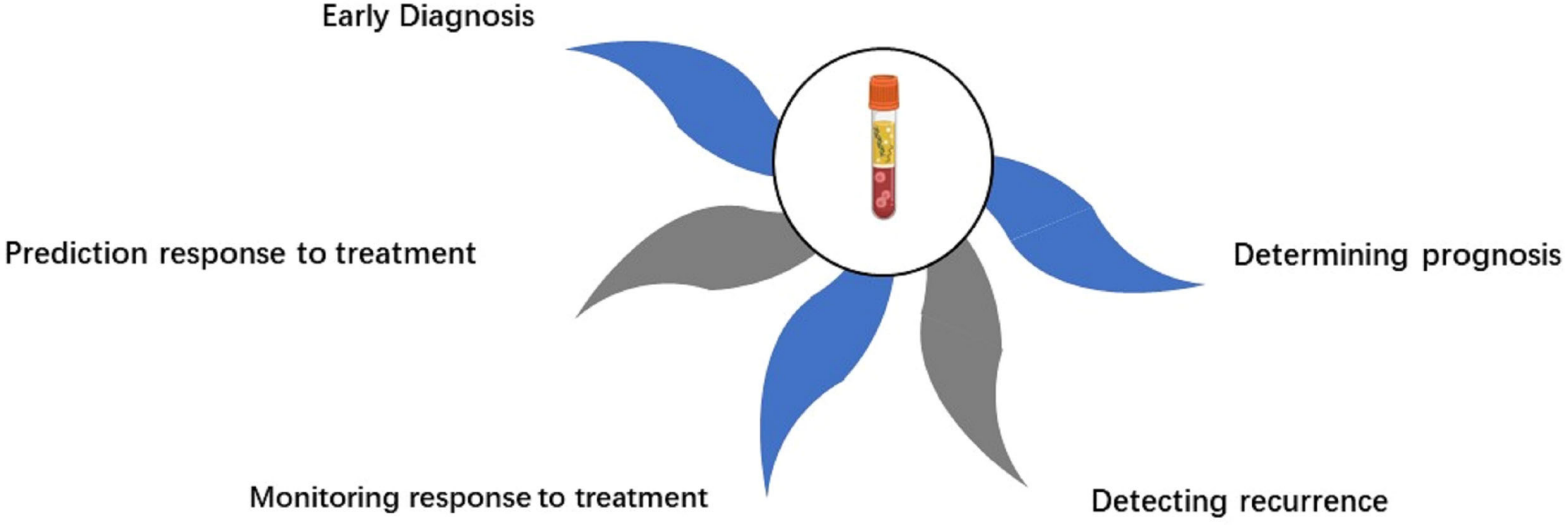
Overview of the five major clinical applications of liquid biopsy in breast cancer.

Liquid biopsy shows promise for improving management of breast cancer and enhancing patient survival, with increasing evidence supporting its potential. Over the past decade, advancements in molecular analysis techniques have enabled widespread application of liquid biopsy for diagnosis, prognosis and predicting treatment response in breast cancer. However, realizing the full potential of liquid biopsy faces several challenges that need to be addressed. **Figure 3** provides a detailed summary of the clinical applications of liquid biopsy components, along with their advantages and disadvantages.

A major challenge with CTCs is their rarity, requiring highly sensitive equipment for detection. The enumeration of CTCs relies on specialized reagents like immunomagnetic beads and automated fluorescence microscopes. However, these techniques have limited sensitivity and accuracy. CTCs analysis is more effective in metastatic patients with higher CTC counts, yet only around half of these patients exhibit positive CTCs (113, 114). Even in metastatic disease, CTCs can comprise less than one cell per billion blood cells. More robust methods are urgently needed to reliably capture, amplify and detect scarce CTCs. Emerging microfluidic and imaging technologies hold promise if challenges with throughput, purity and clinical validation can be overcome. Machine learning methods like deep learning could help improve detection and classification accuracy.

The current challenge with using ctDNA as a biomarker is its low quantity compared to normal cell-free DNA, especially with smaller tumors. CtDNA levels can be as low as 0.01% of total cfDNA. CtDNA is predominantly released from necrotic tumor cells, resulting in longer fragments versus healthy individuals. In contrast, ctDNA from apoptotic cells is shorter at around 133-144bp (115). Size-based isolation can enrich for ctDNA by leveraging its short length. However, this approach may miss longer ctDNA fragments carrying crucial genomic information. Optimized isolation and amplification techniques are needed to comprehensively capture ctDNA diversity. Ultra-deep sequencing could enable detection of rare mutations missed by shallow sequencing. However, this is expensive and bioinformatically challenging currently. Tailored gene panels may provide a balanced approach.

ctDNA analysis could be enhanced by profiling additional hallmark features of cfDNA using integrative approaches. Assessing cfDNA fragmentation patterns, tumor-derived epigenetic signatures, and nucleosome footprints associated with active genes may provide supplementary information to optimize liquid biopsies. This could improve utility for patient selection, risk stratification, and immunotherapy response monitoring. Multiparameter liquid biopsy testing combining circulating biomarkers with cfDNA analysis has shown promise for early cancer detection (116). Similarly, incorporating diverse noninvasive measures, including baseline ctDNA, longitudinal ctDNA changes, and immune cell dynamics, may leverage tumor and immune components to better define molecular response to immunotherapy.

Assessing structural and fragmentation patterns of cfDNA (117, 118), along with tumor-derived epigenetic marks and nucleosome footprints associated with active genes (119), could provide additional features to optimize liquid biopsies. This may improve their utility for patient selection, risk stratification, and monitoring immunotherapy response. Circulating microbiome DNA fragments have also been detected in the blood of melanoma, prostate and lung cancer patients (120). This reveals potential for liquid biopsies to characterize changes in the bacterial microbiome associated with immunotherapy outcomes.

A key limitation is that liquid biopsy may not fully recapitulate tumor heterogeneity, since it samples only some subgroups (121). Multidimensional analysis integrating liquid biopsy data with clinical and radiomic features can help mitigate this. Combining liquid and tissue biopsy may also improve heterogeneity assessment. However, tissue biopsy also has limitations in capturing spatial and temporal heterogeneity. New techniques that can assess tumor evolution are required. Repeated liquid biopsies could help track changes in biomarkers over time. However, standardizing the timing and frequency of longitudinal sampling remains an open question. Mathematical modeling approaches could help optimize longitudinal sampling strategies.

Currently, standardization procedures and calibration methods for liquid biopsy lack consistency. Variations exist in operational workflows and quality control standards employed across different laboratories and studies, resulting in inconsistent results and reduced reproducibility. To address this issue, it is crucial to improve standardization of liquid biopsy practices. One approach is developing consensus guidelines that establish clear protocols and quality control measures for various aspects of liquid biopsy, including sample collection, processing, analysis, and reporting. Such guidelines would provide a standardized framework ensuring consistency and reliability across different laboratories and research settings. Furthermore, implementing external quality assessment (EQA) programs can significantly enhance standardization. EQA programs involve external evaluation and proficiency testing of laboratories, enabling identification of potential errors or variations in testing procedures. By participating in these programs, laboratories can identify areas for improvement and align their practices with established standards.

Collaboration among laboratories is essential to promote standardization in the field of liquid biopsy. Fostering partnerships and sharing best practices allow laboratories to learn from each other’s experiences and work toward harmonizing their approaches. Potential collaborations can include joint research projects, data sharing, and establishing common quality control measures. Through such collaborative efforts, laboratories can shape consensus guidelines and EQA programs that facilitate the standardization of liquid biopsy practices.

Standardization is critical for enhancing the reliability and reproducibility of liquid biopsy results. By improving standardization through laboratory collaborations, consensus guidelines, and EQA programs, liquid biopsy can be implemented consistently in clinical practice. The widespread and uniform utilization of liquid biopsy will only be achieved through improving result consistency. Standardization relies on open collaboration and communication between laboratories to share knowledge and align approaches. By working together, laboratories can promote the standardization needed to move liquid biopsy into routine clinical use.

## Conclusions

Immunotherapy is an highly effective treatment strategy for breast cancer, while there are substantial variations in treatment response among patients. Therefore, it is imperative to identify patient subgroups and enable precision treatment through the use of biomarkers. Liquid biopsies provide a valuable source for assessing various immune-related biomarkers in breast cancer. In this review, we have comprehensively listed and detailed the applications of these immune-related biomarkers. The analysis of PD-L1 on CTCs and exosomes is currently under investigation, while the detection of cfDNA and ctDNA is being utilized with advanced technologies. The value of TMB as an immunotherapy biomarker still requires validation in prospective clinical trials. Currently, there is compelling evidence demonstrating the correlation between genomic markers such as MSI and TCR analysis in the blood of breast cancer patients receiving ICIs therapy, which is associated with treatment efficacy and prognosis.

LB offers several advantages in the context of guiding immunotherapy for breast cancer. One of its key benefits is the ability to obtain multiple and repetitive samples throughout the treatment process, which facilitates effective follow-up and evaluation of treatment response. LB is characterized by its simplicity, rapidity, and minimally invasive nature, making it a convenient tool for monitoring disease progression and therapeutic efficacy.

Immunotherapy, particularly ICIs therapy, can induce distinct alterations in breast cancer. This highlights the importance of determining the optimal timing for extracting biomarkers from LB. By capturing biomarkers at the right time points, clinicians can gain valuable insights into treatment response and tailor therapeutic strategies accordingly. Additionally, different ICIs targeted therapies may require the assessment of specific individual biomarkers or a combination of multiple biomarkers to effectively guide treatment decisions.

While liquid biopsy biomarkers for guiding immunotherapy in breast cancer have not yet been formally recommended in treatment guidelines, current evidence suggests that the non-invasive and feasible nature of LB allows for continuous sampling and longitudinal monitoring. This opens up possibilities for utilizing LB as a valuable tool in guiding the selection of appropriate immunotherapeutic approaches for breast cancer patients.

## Author contributions

JY: Writing – original draft. LQ: Formal Analysis, Writing – original draft. XW: Methodology, Writing – review & editing. XC: Conceptualization, Writing – review & editing. PC: Writing – review & editing. ZY: Writing – review & editing, Supervision. QW: Writing – original draft, Writing – review & editing.
